# Phenotypic and Molecular Characterization of the LOAD2.Plcg2M28L Mouse Model Across Aging and Dietary Conditions

**DOI:** 10.1002/alz70855_104062

**Published:** 2025-12-24

**Authors:** Bruce T. Lamb, Claudia Rangel‐Barajas, Ravi S Pandey, Cynthia M Ingraham, Paul R Territo, Stacey J Sukoff Rizzo, Michael Sasner, Gareth R Howell, Gregory W Carter, Adrian L Oblak

**Affiliations:** ^1^ Indiana University School of Medicine, Indianapolis, IN, USA; ^2^ Stark Neurosciences Research Institute, Indiana University School of Medicine, Indianapolis, IN, USA; ^3^ The Jackson Laboratory, Bar Harbor, ME, USA; ^4^ Indiana University, Indianapolis, IN, USA; ^5^ University of Pittsburgh School of Medicine, Pittsburgh, PA, USA; ^6^ The Jackson Laboratory for Genomic Medicine, Farmington, CT, USA

## Abstract

**Background:**

The LOAD2.Plcg2M28L mouse model presents a valuable tool for investigating late‐onset Alzheimer's disease (LOAD) by incorporating the PLCG2‐M28L variant, a genetic risk factor for developing LOAD. To better understand the molecular and cellular mechanisms associated with this model, we conducted comprehensive phenotyping that included transcriptomic and proteomic analyses, as well as quantitative assessments of microglia, astrocytes, and neurons.

**Method:**

Mice were aged to 4, 12, and 18 months under standard chow and high‐fat diet (HFD) conditions to evaluate the interplay between genetic and environmental factors in disease progression. Our findings reveal distinct age‐ and diet‐dependent changes in brain cellular composition and molecular pathways

**Result:**

At 12 and 18 months, HFD exacerbated neuroinflammatory markers, particularly influencing microglial and astrocytic activation states, while the PLCG2‐M28L variant modulated these responses. Transcriptomic and proteomic profiling identified key alterations in pathways associated with lipid metabolism, immune response, and synaptic function, with significant age‐dependent divergence. Furthermore, neuron counts highlighted progressive neuronal loss exacerbated by HFD, while PLCG2‐M28L carriers displayed resilience to some neurodegenerative phenotypes.

**Conclusion:**

These findings provide novel insights into the role of the PLCG2‐M28L variant in modulating LOAD‐related pathology and underscore the importance of environmental factors such as diet in disease progression. Our results establish the LOAD2.Plcg2M28L model as a robust platform for exploring therapeutic strategies targeting genetic and lifestyle interactions in Alzheimer's disease.

